# Supplementary material for: Family Structure and Family Climate in Relation to Health and Socioeconomic Status for Older Adults: A Longitudinal Moderated Mediation Analysis
Source: Int J Environ Res Public Health. 2022 Sep 19;19(18):11840. doi: 10.3390/ijerph191811840 (PMC9517513; doi:10.3390/ijerph191811840)
Supplement: Supplementary file 1 [file ijerph-19-11840-s001.zip › ijerph-1827234-supplementary.pdf]

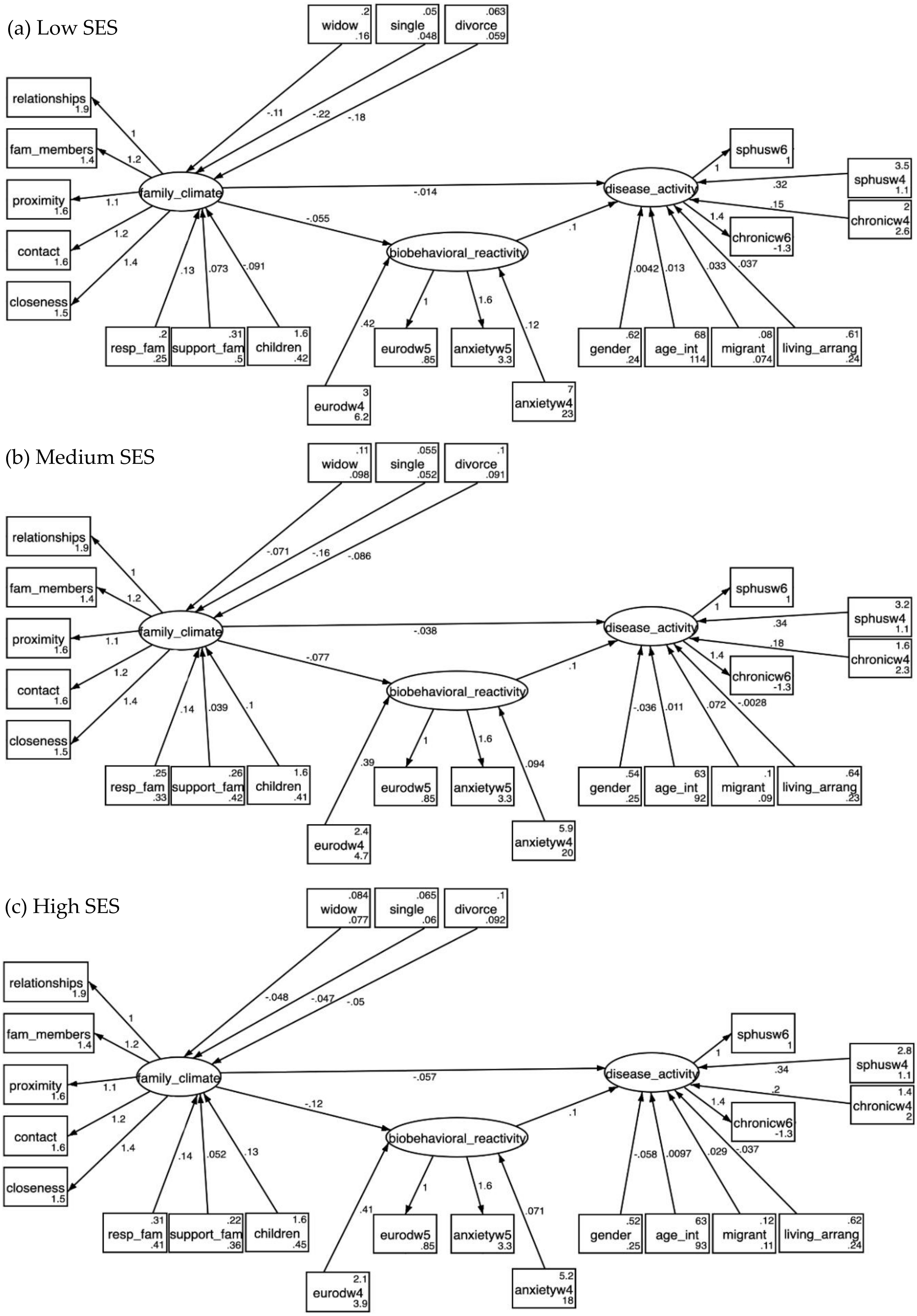

Figure S1. Multigroup structural equation modelling showing the moderated mediation analysis, by group of SES level.

Table S1. Correlation matrix amongst the key manifest variables throughout the three measurement points (n = 29,457).

Variables	1	2	3	4	5	6	7	8	9	10	11	12	13
**Family structure T1**													
1. Widowed	1.000												
2. Divorced	-0.097*	1.000											
3. Single	-0.123*	-0.073*	1.000										
4. Relationship	-0.595*	-0.443*	-0.350*	1.000									
**Family climate T2**													
5. Family members	-0.049*	-0.078*	-0.113*	0.140*	1.000								
6. Proximity	-0.042*	-0.022*	-0.045*	0.069*	0.570*	1.000							
7. Contact	-0.026*	-0.014*	-0.059*	0.058*	0.686*	0.745*	1.000						
8. Closeness	-0.037*	-0.009*	-0.059*	0.060*	0.709*	0.640*	0.744*	1.000					
9. Relationships	0.086*	0.012*	0.034*	-0.084*	0.682*	0.381*	0.453*	0.469*	1.000				
**Biobehavioral React. T2**													
10. Depression T2	0.103*	0.012*	0.002	-0.076*	-0.041*	-0.021*	-0.028*	-0.049*	0.012*	1.000			
11. Anxiety T2	0.073*	0.001*	0.016*	-0.035*	-0.022*	-0.003	-0.020*	-0.044*	-0.006*	0.517*	1.000		
**Disease Activity T3**													
12. Self-rated health T3	0.113*	0.023*	-0.002	-0.061*	-0.032*	-0.053*	-0.064*	-0.100*	-0.013*	0.440*	0.291*	1.000	
13. Chronic diseases T3	0.118*	0.021*	-0.025*	-0.059*	-0.004*	0.003	-0.011*	-0.020*	0.014*	0.292*	0.237*	0.487*	1.000

Note: Pearson correlation coefficients; T1 = wave 4, 2011; T2 = wave 5, 2013; T3 = wave 6, 2015; *coefficients with $p<0.05$

Table S2. Correlation matrix between family structure and the key latent variables throughout the three measurement points (n = 29,457).

Variables	1	2	3	4	5	6	7
**Family structure T1**							
1. Widowed	1.000						
2. Divorced	-0.097*	1.000					
3. Single	-0.123*	-0.073*	1.000				
4. Relationship	-0.595*	-0.443*	-0.350*	1.000			
**5. Family climate T2**	-0.028*	-0.086*	-0.032*	0.525*	1.000		
**6. Biobehavioral React. T2**	0.078*	0.002*	0.012*	-0.020*	-0.026*	1.000	
**7. Disease Activity T3**	0.163*	0.008*	0.017	-0.013*	-0.025*	0.257*	1.000

Note: Pearson correlation coefficients; T1 = wave 4, 2011; T2 = wave 5, 2013; T3 = wave 6, 2015; *coefficients with $p<0.05$
